# Advancing Alzheimer Disease Prediction With Large Language Model–Based Linguistic Feature Analysis: Development and Validation Study

**DOI:** 10.2196/86965

**Published:** 2026-05-28

**Authors:** Ming-Hsia Hsu, San-Yih Hwang, Yi-Hang Tsai, Yun-Chi Chang, Chih-Kuang Liang, Chiung-Yun Chang

**Affiliations:** 1 Department of Information Management, National Sun Yat-sen University, No. 70, Lienhai Rd, Kaohsiung, 804201, Taiwan, +886-7-5252000 ext 4723; 2 Department of Information Systems, Kaohsiung Municipal United Hospital, Kaohsiung, Taiwan; 3 Division of Neurology, Kaohsiung Veterans General Hospital, Kaohsiung, Taiwan; 4 Center for Geriatrics and Gerontology, Kaohsiung Veterans General Hospital, Kaohsiung, Taiwan; 5 Center for Healthy Longevity and Aging Sciences, National Yang Ming Chiao Tung University, Taipei, Taiwan

**Keywords:** alzheimer disease, large language models, linguistic features, prompt engineering, early detection

## Abstract

**Background:**

Alzheimer disease (AD) is a progressive neurodegenerative disorder with rapidly growing global prevalence. Early detection is critical for timely intervention; yet, conventional diagnostic methods remain costly and invasive. Speech-based assessment has emerged as a noninvasive alternative, as AD characteristically impairs linguistic abilities including fluency, coherence, and informational content. Recent advances in large language models (LLMs) offer new opportunities to extract structured linguistic features from transcribed speech for automated AD classification. However, existing LLM-based approaches often lack transparency and clinical interpretability, limiting their adoption in clinical workflows.

**Objective:**

This study aims to investigate the influence of linguistic features extracted from transcribed speech, as analyzed by LLMs, on the accuracy and interpretability of AD prediction.

**Methods:**

We propose a framework that leverages LLMs to analyze linguistic features extracted from transcribed speech for AD classification. Our approach focuses on 4 key aspects, including readability, fluency, richness of detail, and keyword relevance. To enhance classification accuracy, the framework integrates transcript embeddings with feature explanation embeddings, forming a comprehensive linguistic representation. We conducted extensive ablation studies to evaluate the contributions of individual features and benchmarked our framework against existing LLM-driven methodologies through pairwise explainability evaluations. Output stability was assessed across 3 independent pipeline runs. A fully local configuration (Llama 3 8B + nomic-embed-text) was tested to evaluate privacy-preserving deployment feasibility. Explainability was assessed via LLM-based pairwise comparison (Gemini-3.1-flash-lite) against the method of Bang et al across 54 correctly classified cases and by blinded evaluation from 2 neurologists.

**Results:**

The proposed framework achieved a mean precision of 91.52%, a sensitivity of 91.08%, a specificity of 96.29%, and *F*_1_-score of 91.05% across 3 independent runs on the ADReSSo 2021 dataset, outperforming existing LLM-based approaches. A fully-local configuration (Llama 3 8B+nomic-embed-text, requiring no cloud application programming interface access) achieved an *F*_1_-score of 81.58%, demonstrating framework transferability to privacy-preserving deployment environments. Keyword relevance was the most influential feature (*F*_1_-score drop of 13.22 pp when removed). Explainability evaluations showed our method was preferred in 49 out of 54 cases via Gemini-3.1-flash-lite, with human experts preferring our method in 89 of 108 blinded assessments.

**Conclusions:**

These findings highlight that a structured linguistic feature analysis using LLMs provides a robust and interpretable framework for preliminary AD detection. Our approach offers a scalable and accessible solution that bridges artificial intelligence–driven text analysis with clinical applications, supporting early detection of cognitive decline through noninvasive assessment methods.

## Introduction

### Background

Alzheimer disease (AD) is a progressive neurodegenerative disorder that primarily affects older adults. The global prevalence of AD is significant, as the number of people living with dementia is projected to increase from 55 million in 2019 to 139 million by 2050, according to the 2023 World Health Organization (WHO) report [1]. In the United States alone, as of 2024, approximately 6.9 million individuals aged 65 years and older are affected by Alzheimer dementia [2,3]. These alarming figures highlight the critical need for early detection and intervention strategies to improve patient outcomes, optimize treatment strategies, and reduce the burden on health care systems [4].

Early diagnosis of dementia offers substantial benefits, enabling individuals to make informed decisions, access essential services and treatments, and implement preventive measures to maintain safety and quality of life [5,6]. Patients, caregivers, and researchers highly value early detection due to its role in supporting life planning, improving end-of-life care, and facilitating research on disease pathology before advanced neuronal damage occurs [5,7]. Furthermore, early diagnosis may promote lifestyle modifications that could potentially delay or prevent the onset of AD, offering a proactive approach to health management [7].

Although neuroimaging and cerebrospinal fluid biomarkers are considered the gold standard for early detection, their limitations—including high costs, limited availability, and invasive procedures—create accessibility challenges, particularly in resource-limited settings [8,9]. Plasma biomarkers are emerging as promising, noninvasive alternatives that could improve accessibility and reduce wait times for specialized care [8,9]. However, they also face limitations, including variability in results, lower specificity compared to traditional biomarkers, and standardization challenges [10,11]. Additionally, dementia risk scores, such as Cardiovascular Risk Factors, Aging, and Dementia (CAIDE) and the Australian National University Alzheimer Disease Risk Index (ANU-ADRI), provide valuable support for early identification, though ongoing refinement is required for broader clinical application [12].

Integrating routine cognitive assessments in primary care can further enhance early detection rates, promoting proactive dementia management and improved patient outcomes [13-15]. Recent technological developments, including computerized adaptive testing (CAT), enhance the flexibility of digital assessments by tailoring testing experiences based on individual cognitive capabilities [16,17]. Additionally, digital cognitive assessments enable the discovery and longitudinal monitoring of novel digital biomarkers outside traditional clinical settings [18,19]. These tools offer significant advantages over traditional paper-based methods in both clinical practice and research, demonstrating increased sensitivity in detecting subtle cognitive changes that conventional assessments might overlook [18-20]. Digital assessments also provide greater reliability and validity through repeated measurements across multiple days, yielding more consistent intra- and interparticipant data compared to single-time-point traditional assessments [20,21].

The accessibility of digital cognitive assessments is another key advantage, as they allow remote self-administration, reducing the need for in-person clinic visits and improving access for underserved populations [18,22]. Additionally, these digital methods offer high ecological validity by enabling frequent, brief assessments in real-world settings, supporting a patient-centered approach, and generating data that better reflects daily cognitive functioning [20]. Their cost-effectiveness and efficiency make them particularly suitable for large-scale studies and clinical trials requiring scalable solutions [18,22].

AD is increasingly recognized for its impact on cognitive function, particularly memory impairment as an early hallmark. However, language impairment is also a notable early symptom that significantly hinders communication [23]. Studies indicate that integrating language assessments into conventional cognitive evaluations enhances the precision of AD progression prediction [24]. As cognitive decline advances, linguistic tasks become more challenging, highlighting the importance of understanding language dynamics in this patient population. Moreover, the distinct relationship between language deficits and cognitive deterioration underscores the potential of linguistic measures as critical markers for assessing the progression from mild cognitive impairment (MCI) to AD [25]. Recent findings suggest that language assessments can not only identify individuals at greater risk for developing AD but also aid in monitoring the severity of language impairments as the disease progresses [26]. Incorporating language assessment into routine cognitive evaluations could improve early detection strategies, enabling targeted interventions designed to help maintain communication abilities. Thus, recognizing and addressing language impairments in early AD stages could play a pivotal role in patient care and management.

In recent years, deep learning-based approaches have gained traction for automating feature extraction by learning complex representations from speech data. Various speech embeddings, such as VGGish, X-vectors, and Wav2Vec [27], along with language embeddings like Bidirectional Encoder Representations from Transformers (BERT), Robustly Optimized BERT Pretraining Approach (RoBERTa), and GPT, have been used to capture rich acoustic and linguistic information for AD detection [28-31]. Moreover, studies demonstrate that combining acoustic and linguistic features enhances AD detection performance by integrating multiple aspects of speech and language affected by cognitive decline [32-34].

Despite the strong performance of deep learning-based approaches, they face significant challenges regarding explainability and interpretability. A systematic review conducted by Shi et al [31] analyzed 72 studies and found that most only provided selective examples for their deep learning models, with very few explicitly addressing explainability. However, in clinical settings, there is a growing need for simpler yet interpretable methods for preliminary AD screening. Recent developments in large language models (LLMs) have demonstrated promise in analyzing speech transcripts from the Cookie Theft picture description task, with studies reporting accuracies of 80.3% using GPT-3 [35] (OpenAI). Notably, Bang et al [36] developed a method incorporating LLM-driven explainability, achieving 85.92% accuracy using GPT-4 (OpenAI).

In this research, we expand on the explainability capabilities of LLMs and systematically refine their prompt design. We guide the LLM to evaluate 4 key linguistic dimensions—readability, fluency, richness of detail, and keyword relevance—before making a diagnosis. This approach enhances both accuracy and interpretability. Our proposed framework also introduces a structured prompt template that directs LLMs in systematic linguistic analysis. Our design includes (1) expert role definition as a speech therapist, (2) specific task instructions for feature analysis, (3) standardized feature definitions, and (4) exemplar demonstrations.

Using Whisper (OpenAI) for speech-to-text conversion and GPT-4 with our specialized prompt template, our framework addresses a binary classification task**—**distinguishing individuals with AD from cognitively control normal (CN)—and achieved around 91% precision and recall, with 96% specificity on the ADReSSo 2021 dataset, demonstrating that structured linguistic analysis alone can support reliable preliminary AD screening. Key contributions of this work include:

1. Proposing a novel framework integrating LLM-based linguistic analysis with structured feature evaluation and prompt engineering for generating explainable AD diagnosis.
2. Developing an AD classifier that incorporates both transcript content and LLM-explained features to enhance prediction accuracy.
3. Providing empirical evidence of stable and competitive diagnostic accuracy (*F*_1_-score=91.05%; sensitivity=91.08%; specificity=96.29%) on the ADReSSo 2021 Challenge dataset using speech transcripts from the Cookie Theft picture description task [36].
4. Demonstrating superior explainability through a structured multifeature framework, significantly outperforming an existing approach in diagnostic reasoning, evidence support, and clinical insight, winning 49 out of 54 pairwise evaluations via Gemini-3.1-flash-lite.

### Related Work

Before the advent of ChatGPT (OpenAI) in late 2022, most deep learning-based research for AD detection predominantly used BERT as the underlying machine learning framework. In these studies, linguistic features and language embeddings served as inputs to BERT. Subsequent studies adopted more advanced artificial intelligence (AI) models, such as GPT-3 and GPT-4, for AD detection. Below, we review these studies and highlight their strengths and weaknesses.

### AD Detection With BERT

BERT is a transformer-based pretrained language model introduced by Google researchers in 2018 [37]. It represents text as a sequence of vectors, which can then be used to train a classification model. The application of BERT for direct transcript embedding has shown promising results in several studies. For instance, Padhee et al [38] applied BERT to raw transcriptions, achieving an *F*_1_-score of 80% for classifying patients with AD, MCI, and, while Rohanian et al [39] highlighted BERT’s adaptability to variations in transcription quality.

For feature-enriched embedding approaches, researchers have enhanced BERT’s capabilities by incorporating additional linguistic features. Mahajan and Baths [40] improved multimodal classification by integrating lexical diversity and syntactic complexity features with BERT embeddings. Qiao et al [41] focused on model explainability by combining fluency and disfluency features, while Yuan et al [42] examined semantic similarity and information density as complementary inputs to increase diagnostic accuracy.

Despite these successes, BERT-based methods face key challenges; direct embedding approaches lack direct clinical explainability, while feature-enriched methods introduce additional complexity and require domain expertise.

### AD Detection With Advanced LLM

The emergence of more advanced AI models, such as GPT-3 and GPT-4, has transformed AD detection through innovative approaches to linguistic feature extraction and analysis. Compared to earlier models like BERT, these LLMs excel at classification tasks, even with minimal or no additional training data, while also providing textual explanations.

Agbavor and Liang [35] pioneered the use of GPT-3 for dementia prediction from spontaneous speech, leveraging text embeddings to capture semantic meaning and achieving 80.3% accuracy in distinguishing between patients with AD and CNs. Wang et al [43] expanded this research by exploring GPT-4’s capabilities for MCI screening, analyzing linguistic indicators via standardized prompts, and achieving 77.3% sensitivity and 83.3% specificity.

Bang et al [36] introduced a novel methodology by using GPT-4 for speech fluency evaluation, integrating AI-generated opinions with original text, and achieving 85.92% accuracy and 94.44% specificity. Additionally, Balamurali and Chen [44] conducted a comparative analysis of multiple LLMs in a zero-shot learning context, highlighting both the potential and the limitations of LLM technology in clinical settings at the time of this writing.

These developments illustrate the progression from basic text analysis to sophisticated diagnostic tools, while also emphasizing the need for standardized prompt engineering and clinical validation. This growing body of research suggests that well-structured LLM approaches could provide valuable support for preliminary AD screening while maintaining interpretability for health care professionals.

However, these LLM-based approaches face two major limitations:

1. Lack of transparency and interpretability: many LLM-driven approaches do not clearly specify the evaluation process. For instance, while Bang et al [36] used GPT-4 to assess speech fluency, they did not define which fluency aspects were being measured. Similarly, Agbavor and Liang’s [35] approach using GPT-3 embeddings lacked clarity regarding the specific semantic features captured.
2. Absence of a structured analysis framework: previous studies varied significantly in their methodological approaches, lacking a standardized framework for LLM-based analysis. Although Balamurali and Chen [44] specified multiple linguistic aspects for evaluation, their LLM-based approach remained exploratory, without establishing scoring criteria or standardization. This lack of methodological structure limits the clinical applicability of these models.

These limitations underscore the need for a systematic and interpretable approach to LLM-based AD detection, one that integrates structured feature evaluation with explainability. In this work, we address these challenges by developing a framework that enhances transparency, interpretability, and clinical relevance.

## Methods

### Overview

Before introducing our methodology, we first describe the dataset used in this research. Our approach begins with extracting AD-relevant linguistic features using an LLM. We meticulously design prompts to guide the LLM in analyzing linguistic patterns associated with AD. The resulting feature explanations, along with their corresponding transcripts, are then used to construct an AD classifier. Detailed descriptions are provided in the following subsections.

### Dataset

The primary dataset used in this study is the ADReSSo 2021 Challenge corpus [45], derived from DementiaBank [46]. This dataset contains audio recordings of participants describing the Cookie Theft picture from the Boston Diagnostic Aphasia Examination (BDAE) [47], a standardized task widely used in cognitive assessments. The dataset has been carefully balanced to address common demographic biases in medical datasets, ensuring matched distributions of age and gender between groups.

The dataset comprises 166 training samples, including 87 AD cases and 79 CNs, and 71 test samples, including 35 AD cases. All audio recordings underwent preprocessing to ensure consistency. The dataset’s balanced structure mitigates the common issue of group imbalance in clinical datasets, making it particularly suitable for developing and evaluating AD classification models.

### The Framework

**Figure 1.**
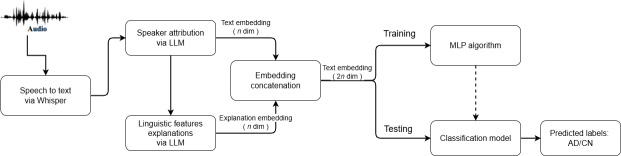
The framework of the proposed method that incorporates both transcript and its feature explanations for Alzheimer disease (AD) prediction. AD: Alzheimer disease; CN: control normal; LLM: large language model; MLP: multilayer perceptron.

The overall framework of our AD prediction method is illustrated in Figure 1.

The process consists of the following steps.

1. Speech-to-text conversion performed by Whisper.
2. Speaker attribution to extract the participant’s description from the transcript using an LLM-based approach.

The first 2 preprocessing steps are described in the Preprocessing subsection. The examiner-free transcript is then analyzed by an LLM, which generates feature-based explanations across 4 linguistic categories. These features are detailed in the AD Related Linguistic Feature section. Both the transcript and its corresponding explanations are converted into *n*-dimensional vector representations. These embeddings are then concatenated into a 2*n*-dimensional vector, integrating both semantic content and feature-based assessment. A multilayer perceptron (MLP) classifier processes the final representation to classify participants into AD or CN groups.

### Preprocessing

Our preprocessing pipeline consists of 2 essential steps to derive transcripts from the dataset.

1. Speech-to-text conversion: all audio recordings were transcribed using the Whisper automatic speech recognition (ASR) system [48].
2. Speaker attribution: since the raw transcripts contain dialogues between examiners and participants, this step aims to isolate the participant’s descriptions by removing examiner interventions (eg, “What’s happening in that picture?”). We compared 2 methods: an LLM-based speaker attribution method and a conventional speaker diarization method using PyAnnote (pyannoteAI). Experimental results on the ADReSSo 2021 dataset show that the LLM-based speaker attribution method yields better performance (*F*_1_-score=0.82) compared to the conventional speaker diarization method (*F*_1_-score=0.92). We observe that the speaker diarization method using PyAnnote [49] was prone to failure or speaker misidentification in low-volume recordings and cases where examiner and participant voices were acoustically similar, resulting in incomplete or contaminated transcripts. The LLM-based method, by contrast, leverages semantic content to reliably identify and remove examiner turns regardless of audio quality, preserving richer participant descriptions. Based on these results, the LLM-based approach was adopted for subsequent analyses. Readers are referred to Multimedia Appendix 1 for the detailed prompt design.

### AD-Related Linguistic Feature Analysis With LLM

This study identifies 4 key linguistic features—readability, fluency, richness of detail, and keyword relevance—as critical for detecting AD from speech. These features were derived from a comprehensive review of linguistic studies and correspond to established categories in speech and language processing, including syntactic, semantic, lexical, disfluency, and pragmatic features. Each feature captures a distinct aspect of language impairment observed in AD, offering a structured framework for assessing cognitive decline. Table 1 summarizes these features, their relevant linguistic categories, primary focus, quantification metrics, and key references.

**Table 1.** Linguistic features for Alzheimer disease (AD) detection.

Feature	Relevant linguistic category	Core focus	Metrics	Key references
Readability	Syntactic and lexical	Evaluating syntactic complexity, lexical diversity, and discourse coherence to reflect the organization of speech.	Lexical diversity (Type-Token Ratio; TTR) Syntactic complexity (dependency parsing) Discourse coherence (Coh-Metrix indices)	[38,40-42,50,51]
Fluency	Disfluencies	Measuring smoothness and flow of language, including hesitations, filled pauses, and repetitions.	Pause frequency and duration Speech rate Self-repair rates	[39,40,52-54]
Richness of detail	Semantic and pragmatic	Assessing the density and specificity of meaningful content in descriptions.	Proportion of information-bearing nouns and verbs Content density (Latent Semantic Analysis) Ratio of semantically empty words	[42,55,56]
Keyword relevance	Semantic	Evaluating alignment of spoken content with predefined key elements in a given context (eg, the Cookie Theft image).	Frequency of target keywords Semantic similarity (cosine similarity and topic modeling) Neural attention weights for key terms.	[54,57,58]

Readability encompasses syntactic complexity, lexical diversity, and discourse coherence, all of which are essential for evaluating the organization and comprehensibility of speech. Studies indicate that patients with AD often produce grammatically simplified sentences, shorter sentence segments, and less coherent discourse, reflecting a decline in their ability to construct complex and information-dense narratives [40,41,50]. Traditional readability assessments rely on dependency parsers and Coh-Metrix indices, but LLMs provide a holistic alternative by integrating syntactic and lexical patterns into a unified framework [42,51].

Fluency captures the smoothness and temporal flow of speech, focusing on pauses, hesitations, and repetitions. Patients with AD frequently exhibit disfluencies due to word retrieval challenges and sentence formulation difficulties, which signal cognitive decline [55,56]. Common metrics include speech rate, pause frequency, and self-repair rates. LLMs enhance fluency analysis by automatically detecting patterns within transcripts, offering a scalable and nuanced approach to fluency evaluation [40,52].

Richness of detail refers to the density of meaningful and specific information within a description. Studies indicate that patients with AD tend to provide fewer information-bearing propositions, relying on vague or semantically empty words [55,56]. This reflects impairments in semantic memory and access to stored knowledge. Metrics such as latent semantic analysis and content density analysis quantify these deficits. LLMs enable dynamic evaluations by assessing the use of descriptive language and narrative coherence, complementing traditional feature extraction methods [42].

Keyword relevance evaluates the degree to which spoken content aligns with predefined key elements of a context or scenario, such as the Cookie Theft picture used in our study. The patients with AD often omit critical objects or actions, reflecting impairments in lexical retrieval and semantic memory [55,56]. Neural attention models have shown that diagnostically relevant keywords (eg, “sink,” “water,” and “cookie”) appear significantly less frequently in the descriptions of patients with AD[57]. LLMs improve keyword analysis by leveraging attention mechanisms to quantify the inclusion and contextual relevance of key terms [58].

These 4 linguistic features collectively address syntactic, lexical, semantic, and pragmatic aspects of AD-related language impairments. Traditional methods rely heavily on manual feature engineering and domain-specific tools, whereas LLMs provide an automated and holistic approach by integrating structured, semantic, and pragmatic analyses via carefully designed prompts. This LLM-driven approach provides a scalable and interpretable method for analyzing speech, aligning with recent advancements in biomedical informatics for dementia diagnosis [55,56,59].

### Prompt-Template Design

**Figure 2.**
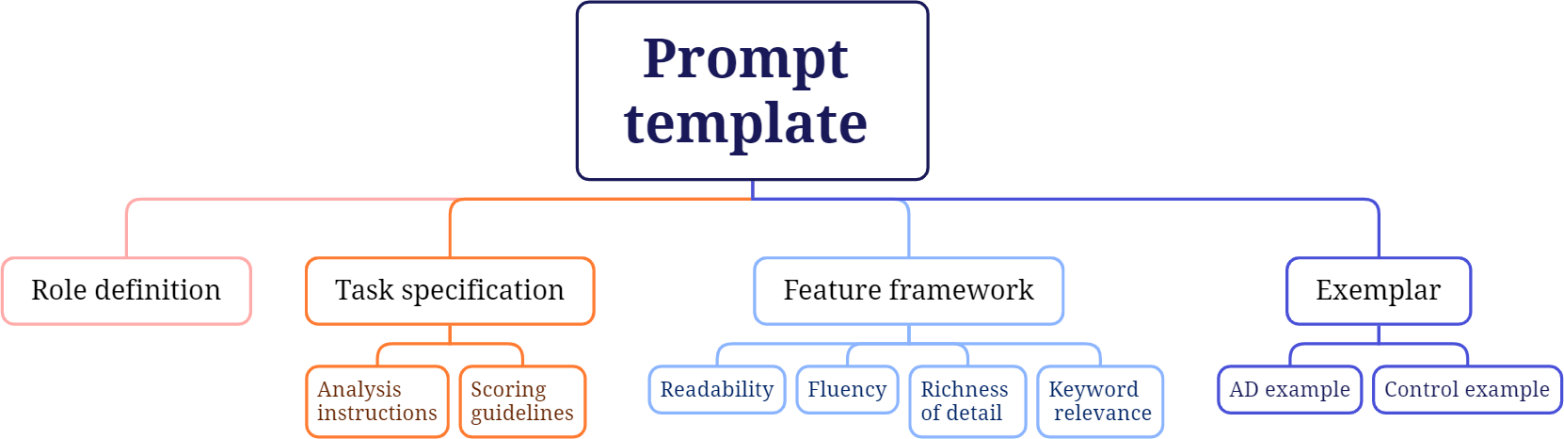
The hierarchical structure of the prompt template, illustrating the relationships between components and their respective subelements. AD: Alzheimer disease.

We developed a structured prompt template to facilitate consistent and comprehensive LLM-based analysis of Cookie Theft picture descriptions. The complete template is provided in Multimedia Appendix 1. The template is designed hierarchically, consisting of 4 key components (illustrated in Figure 2).

The 4 key components are:

1. Role definition: establishes the expert context to ensure that the LLM provides clinically relevant responses [60]. This component positions the LLM as a speech and language therapist with expertise in identifying language dysfunctions in individuals with cognitive impairment.
2. Task specification: provides analysis instructions and scoring guidelines to ensure structured evaluations. The analysis instructions explain that language dysfunctions in AD often arise from compromised semantic and pragmatic processing abilities and that the Cookie Theft picture description task is specifically designed to assess cognitive function and memory. The scoring guidelines direct the LLM to evaluate each feature on a standardized 1‐7 scale, where higher scores indicate better cognitive function. These guidelines emphasize the importance of providing detailed explanations and specific evidence from the transcript to support each feature assessment.
3. Feature definition: features to be evaluated include readability (syntactic complexity and comprehensibility), fluency (speech smoothness and coherence), richness of detail (information density and specificity), and keyword relevance (inclusion of essential elements from the Cookie Theft picture). Each feature definition includes brief descriptions of how these linguistic elements typically manifest in patients with AD compared to healthy individuals.
4. Exemplar demonstrations: the template includes 2 contrastive examples to calibrate the analysis, including a control example, generated from a participant with high cognitive function, and an AD example, produced by a participant with AD. The control example consists of a detailed, well-structured description demonstrating high scores across all features, whereas the AD example involves a description showing typical patterns of cognitive decline, characterized by fragmented expression and limited detail. Refer to Multimedia Appendix 1 for the full prompt and an example of AD-related linguistic feature analysis generated by the LLM.

### Ethical Considerations

This study uses the publicly available ADReSSo 2021 dataset from the DementiaBank corpus, which does not involve direct patient contact or new clinical data collection. Institutional Review Board (IRB) approval (25-CT1-02[241015-2]) was obtained through an expedited review at Kaohsiung Veterans General Hospital, covering the methodological framework development and serving as a prerequisite for the associated clinical pilot study. No additional ethics approval was required for use of the publicly available dataset.

## Results

### Configuration

We developed a multicomponent framework for AD detection, with each component specifically configured. We adopted GPT-4 [61] as the large language model in our experiments. For the embedding process, we used OpenAI’s text-embedding-ada-002 [61] model to generate embeddings from both transcript content and feature explanations. Each component produced a 1536-dimensional vector, resulting in a concatenated 3072-dimensional vector for the subsequent classification task. For the classification model, we implemented a 2-layer MLP architecture. The first layer transformed the 3072-dimensional input into 512 hidden units with rectified linear unit activation and dropout (rate=0.1), followed by a second layer outputting binary classification probabilities. The model was trained using the Adam optimizer [62], a batch size of 8, a learning rate of 0.01, and ran for 50 epochs. We used 5-fold cross-validation for model evaluation, with each fold maintaining balanced AD/Control ratios, using the 176 training samples of the ADReSSo 2021 dataset. Cross-entropy loss was used as the optimization criterion.

### Performance Results

The proposed framework was benchmarked against 3 comparative approaches that used LLMs for AD detection from speech transcripts using the ADReSSo 2021 dataset.

We compared against 2 published methods, including Agbavor and Liang [35], who derived semantic representations using GPT-3 embeddings, and Bang et al [36], who combined GPT-4-based fluency assessments with transcript embeddings.

To assess output stability under the inherent stochasticity of LLM-based feature generation, we conducted 3 independent runs of the full pipeline using identical configurations and report the mean results, which are shown in Table 2. The mean *F*_1_-score of 91.05% and mean specificity of 96.29% demonstrate consistent performance across runs. Importantly, the mean *F*_1_-score exceeds that reported in Bang et al [36] (85.80%) by 5.25 percentage points, confirming that the framework maintains its advantage over existing methods even under stochastic variation.

**Table 2.** Performance comparison of different LLM^a^-based approaches for AD^b^ detection.

Research	LLM	Approach	*F*_1_-score%	Accuracy%	Precision%	Sensitivity%	Specificity%
Agbavor.and Liang (2022) [35]	GPT3	Semantic embeddings	—^c^	80.3	80.6	80.6	—
Bang et al (2024) [36]	GPT4	Fluency assessment + embeddings	85.80	85.92	86.94	85.92	94.44
Our proposed framework	GPT4	Multifeature embeddings + MLP^d^	91.05	91.08	91.52	91.08	96.29
Our proposed framework Llama 3 8B+nomic-embed (fully local)	Llama 3	Multifeature embeddings + MLP (fully local, no cloud API^e^)	81.58	81.69	82.30	81.69	88.89

a LLM: large language model.

b AD: Alzheimer disease.

c Not applicable.

d MLP: multilayer perceptron.

e API: application programming interface.

To evaluate feasibility for privacy-sensitive clinical environments where cloud-based application programming interfaces may not be permissible, we tested a fully local configuration using Llama 3 (8B) [63] for feature extraction and nomic-embed-text [64] for embedding generation, requiring no cloud application programming interface access. This configuration achieved an *F*_1_-score of 81.58% and a specificity of 88.89%, as shown in the last row in Table 2. The performance gap relative to the proposed framework’s mean (9.47 pp=91.05% mean *F*_1_-score) highlights the capability limitations of the fully local configuration, specifically the LLM and embedding model used, namely, Llama 3 (8B) and nomic-embed-text. Note that in the future, as more capable open-source LLMs and embedding models mature, the performance of fully local deployments is expected to improve substantially.

Compared to previous LLM-based methods, our framework demonstrates substantial improvements. The enhancement over Bang et al [36], which also uses GPT-4, is particularly notable, with improvements of approximately 5 percentage points in accuracy and *F*_1_-score and 1.9 percentage points in specificity. This improvement can be attributed to our systematic integration of 4 distinct linguistic features (readability, fluency, richness of detail, and keyword relevance) and the combination of transcript and feature explanation embeddings.

The mean specificity (96.29%) achieved by our framework is particularly significant in clinical contexts, as it indicates strong capability to correctly identify non-AD cases, thereby reducing false positives in preliminary screening scenarios. This specificity exceeds Bang et al [36] (94.44%), highlighting the clinical utility of our structured multifeature approach for reducing unnecessary follow-up.

### Ablation Study

To assess the contribution of individual components in our framework, we conducted comprehensive ablation experiments across 3 key aspects, namely the impact of individual features, the effect of embedding combinations, and the consequence of prompt design. All ablation experiments were conducted using a single fixed pipeline run to ensure that observed performance differences reflect the contribution of each component rather than run-to-run stochastic variation. The results are shown in Table 3.

**Table 3.** Ablation study results: Impact of individual linguistic features on model performance.

Configuration	*F*_1_-score	Accuracy	Precision	Sensitivity	Specificity
Full model	91.52	91.55	92.07	91.55	97.22
Without readability	85.90	85.92	86.02	85.92	88.89
Without fluency	88.73	88.73	88.73	88.73	88.89
Without richness of detail	84.50	84.51	84.53	84.51	86.11
Without keyword relevance	78.3	78.87	81.87	78.87	94.44

### Individual Feature Impact

The full model (*F*_1_-score=91.52%) in Table 3 represents the result of the same fixed run used across all ablation conditions, providing a controlled baseline for comparing the effect of each component removal. We evaluated the importance of each linguistic feature by removing them one at a time, with the results shown in Table 3. Our findings show that all features contributed greatly to the model’s performance, with keyword relevance being particularly crucial (dropping from 91.52% to 78.30% *F*_1_-score when removed). The other features showed smaller yet still notable impacts: readability (*F*_1_-score=85.90%), fluency (*F*_1_-score=88.73%), and richness of detail (*F*_1_-score=84.50%).

### Embedding Combination Effect

Our approach uses both transcript and feature embeddings. We intend to investigate the impact of each embedding type separately. Our findings, shown in Table 4, reveal that the use of both text and feature embeddings significantly outperformed those using either type of embeddings alone. The full model achieved a 91.52% *F*_1_-score, while using only text embeddings or feature embeddings yielded 77.44% and 57.70%, respectively. This demonstrates the complementary nature of these representations.

**Table 4.** Impact of different embedding configurations and prompt structures on model performance.

Configuration	*F*_1_-score	Accuracy	Precision	Sensitivity	Specificity
Full model	91.52	91.55	92.07	91.55	97.22
Embedding configuration					
Text embedding only (1536 dim)	77.44	77.46	77.53	77.46	80.56
Feature embedding only (1536 dim)	57.70	57.75	57.74	57.75	61.11
Prompt structure					
Without few-shot examples	85.86	85.92	86.36	85.92	91.67
Without feature definitions	85.80	85.92	86.94	85.92	94.44
Without AD^a^ relation descriptions	85.90	85.92	86.02	85.92	88.89

a AD: Alzheimer disease.

### Prompt Design Impact

Our approach involves a structured prompt with several key elements. In this experiment, we analyzed performance changes after removing specific prompt components. The results, as detailed in Table 4, show that the full structured prompt template significantly enhanced performance (*F*_1_-score 91.52%), while removing few-shot examples (85.86%), feature definitions (85.80%), or AD relation descriptions (85.90%) led to performance declines.

## Discussion

### Principal Findings

To assess the explainability of our structured prompt design, we conducted a pairwise comparison with Bang et al [36], which also provides diagnostic explanations for each transcript. Pairwise comparison is a well-established evaluation method in which 2 alternatives are directly compared based on specific criteria. The comparison focused on 54 cases where both methods correctly classified the samples, ensuring a fair assessment that isolates the evaluation of explanation quality. The feature explanations used in this comparison were drawn from a single randomly selected pipeline run, ensuring that the evaluation reflects a representative output rather than a curated best-case result.

We used Gemini-3.1-flash-lite [65] as an independent judge, instead of GPT-4 used in our method, to avoid self-preference, following recent natural language processing practices. This approach enables a cost-efficient, systematic, and consistent evaluation across multiple samples, making it particularly well-suited for comparing natural language explanations [66]. We evaluated the explanations using 5 key criteria derived from explainable AI literature [67-71], namely diagnostic connection, which assesses how well linguistic features are linked to AD diagnosis [67]; evidence support, which examines the concrete evidence provided from the transcript [68]; clinical insight, which evaluates the value of insights for clinical assessment [69]; feature coverage, which measures the comprehensiveness of linguistic feature analysis [70]; and actionable information, which assesses the usefulness for health care professionals [71].

Table 5 reports the numbers of wins for the method of Bang et al [36], wins for our method, and ties, as evaluated by Gemini-3.1-flash-lite. It shows that our method significantly outperformed the approach of Bang et al [36] across all criteria, with our method preferred in 49 out of 54 cases. The most notable difference appeared in feature coverage, where our approach received 52 out of 54 preferences. These results highlight the strength of our structured prompt design, which systematically addresses multiple linguistic dimensions (readability, fluency, richness of detail, and keyword relevance), providing a more comprehensive and clinically relevant explanation compared to fluency-focused approaches.

**Table 5.** Pairwise comparison results via Gemini-3.1-flash-lite.

Criteria	Wins for the method by Bang et al [36]^a^	Wins for our method^a^	Ties
Diagnostic connection	3	31	20
Evidence supports	16	38	0
Clinical insight	9	45	0
Feature coverage	2	52	0
Actionable information	5	49	0
Overall winner	5	49	0
Confidence level			
High	9.26	90.74	0.00
Medium	0.00	0.00	0.00
Low	0.00	0.00	0.00

a Values represent counts out of 54 pairwise comparisons.

**Figure 3.**
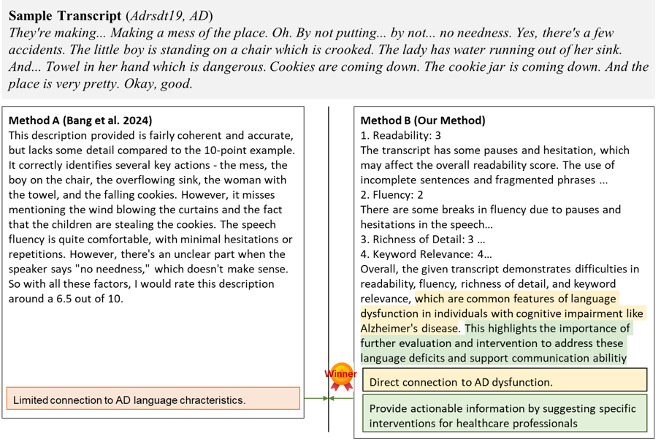
Comparison of explanation methods. AD: Alzheimer disease.

Figure 3 illustrates a representative case comparison between the 2 explanation methods. In this example, the method of Bang et al [36] provides a general assessment of fluency, lacking structured analysis or explicit links to AD symptomatology. By contrast, our method offers a systematic evaluation across multiple linguistic features, with numerical scoring and specific observations linked to language dysfunction in AD.

### Human Expert Evaluation

To address potential concerns regarding LLM-based evaluation bias, we conducted an independent human evaluation involving 2 neurologists from Taiwan medical centers. The experts were presented with randomized explanations from both methods for the same 54 cases in a blinded fashion, without knowledge of which method generated each explanation.

Evaluator A assigned preference to our method in 53 (98.1%) cases and to the method of Bang et al [36] in 1 case, with no tied evaluations. Evaluator B assigned preference to our method in 36 (66.7%) cases and to the method of Bang et al [36] in 9 cases, with 9 tied evaluations. Combined results showed 89 total preferences for our method (53+36) versus 10 for the method of Bang et al [36] (1+9), with 9 ties. Such a difference between human annotators is not a surprise, as several research studies have demonstrated that human judgments are more diverse and variable [72-74]. Nevertheless, these results demonstrate strong interevaluator agreement favoring our structured multifeature approach, with Evaluator A showing near-unanimous preference and Evaluator B displaying greater variability while maintaining overall preference for our method.

The convergence between automated and human expert evaluations strengthens the validity of our explainability assessment and demonstrates that our structured multifeature approach provides substantially more interpretable and clinically relevant explanations for AD detection from speech.

Figure 3 illustrates a representative case comparison between the 2 explanation methods. In this example, the method of Bang et al [36] provides a general assessment of fluency, lacking structured analysis or explicit links to AD symptomatology. By contrast, our method offers a systematic evaluation across multiple linguistic features, with numerical scoring and specific observations linked to language dysfunction in AD.

Analysis of representative cases reveals that our structured approach explicitly connects linguistic observations to cognitive impairment patterns typical of AD. For example, in case “Adrsdt19,” our method identified specific language disruptions such as hesitations, repetitions, and fragmented expressions, directly linking these to potential cognitive decline. In contrast, the approach of Bang et al [36] often described language performance without establishing clear connections to AD-related impairments.

Note that LLM-based evaluation metrics should be interpreted with appropriate care; the convergence between Gemini-3.1-flash-lite and human expert assessments collectively strengthens the validity of our explainability findings.

To provide empirical support for feature selection, we conducted 2-tailed independent-samples *t* tests comparing LLM-derived feature scores between AD (n=122) and CN (n=115) groups (RStudio; Posit Software and R v4.5.0; R Foundation for Statistical Computing [75]). The feature scores were obtained from a single randomly selected pipeline run, consistent with the approach used for the pairwise explainability evaluation. As shown in Table 6, all 4 features showed highly significant group differences (all *P*<.001; df=235) with large effect sizes (Cohen *d* range: 1.11‐1.19). The largest mean difference was observed for richness of detail (AD: 3.42 vs CN: 5.04; *Δ*=1.62), consistent with reduced informational specificity in AD speech. These findings independently validate the selection of the 4 features for the classification framework.

**Table 6.** *T*-test results comparing linguistic feature scores between Alzheimer disease (AD) and control normal (CN) groups.

Linguistic feature	AD^a^ group, mean (SD)	AD, median (IQR)		CN^b^ group, mean (SD)	CN, median (IQR)		*P* value
Readability	4.00 (1.47)	4 (3-5)		5.49 (1.12)	6 (5-6)		<.001^c^
Fluency	3.68 (1.37)	4 (2-5)		5.23 (1.24)	6 (5-6)		<.001^c^
Richness of detail	3.42 (1.48)	3 (2-4)		5.04 (1.44)	5 (4-6)		<.001^c^
Keyword relevance	3.84 (1.61)	4 (3-5)		5.54 (1.43)	6 (5-7)		<.001^c^

a AD: Alzheimer disease.

b CN: control normal.

c Degrees of freedom (df)=235.

We acknowledge that certain non-LLM state-of-the-art methods have reported higher diagnostic precision. For instance, Liu et al [76] achieved an accuracy of 97.18% and an *F*_1_-score of 97.09% on the same dataset by using a Mozilla Deep Speech ASR and BERT-based pipeline. While such deep-learning models excel in raw performance, they often function as ’black boxes’ with limited clinical interpretability. In contrast, our framework prioritizes explainable AI (XAI) by generating structured linguistic evidence across 4 dimensions. Our results demonstrate that while achieving an accuracy of 91.08%, the primary value of this work lies in providing transparent, actionable insights that are essential for clinical trust and diagnostic reasoning.

These findings suggest that our structured multifeature prompt design provides substantially more interpretable explanations for AD detection from speech. Enhanced explainability can improve clinical trust in AI-assisted diagnosis, potentially facilitating adoption in health care settings where transparency is essential. Furthermore, the detailed linguistic breakdowns generated by our method could support more targeted intervention strategies, enabling clinicians to focus on specific linguistic deficits observed in patients.

### Conclusion

Our structured LLM-based framework, which leverages 4 key linguistic features (readability, fluency, richness of detail, and keyword relevance), achieved 92% precision and 97% specificity in detecting AD from speech transcripts. While further clinical validation is needed, this work demonstrates that well-structured linguistic analysis using LLMs can provide a reliable and explainable method for preliminary AD screening. Our framework offers an accessible tool for early detection of cognitive decline, potentially reducing barriers to timely diagnosis. The high performance and interpretable outputs of our model suggest promising directions for integrating AI-assisted cognitive assessment into clinical practice. Particularly in early screening stages, where accessible, noninvasive assessment tools are most valuable, our method could serve as an effective complement to traditional diagnostic approaches.

Despite presenting key innovations in AD detection through structured linguistic analysis, our study has several limitations. The ADReSSo 2021 dataset, while balanced, may not fully capture the linguistic variability found across the patient with AD population. Additionally, our method is currently designed for English-specific transcripts, requiring multilingual adaptation for broader clinical applicability. Although the ADReSSo 2021 dataset is demographically balanced with age and gender matched between groups, broader clinical deployment may require demographic-adjusted scoring baselines to account for variables such as education level, native language, and cultural background; this remains a direction for future work. Further studies are needed to assess how AI-based assessments align with real-world medical diagnoses and clinical outcomes. Furthermore, our reliance on transcript-based analysis introduces potential ASR errors, suggesting future work should incorporate direct audio processing techniques.

Despite these challenges, our findings demonstrate that structured LLM-based linguistic assessment provides a scalable, interpretable tool for early AD detection. This approach effectively bridges the gap between AI-based text analysis and real-world clinical applications, offering a step forward in leveraging AI for cognitive health monitoring.

## Supplementary material

10.2196/86965 Multimedia Appendix 1 Large language model prompt templates for speaker attribution and linguistic feature analysis.

## Abbreviations

AD: Alzheimer disease
AI: artificial intelligence
ANU-ADRI: Australian National University Alzheimer Disease Risk Index
ASR: automatic speech recognition
BDAE: Boston Diagnostic Aphasia Examination
BERT: Bidirectional Encoder Representations from Transformers
CAIDE: Cardiovascular Risk Factors, Aging, and Dementia
CAT: computerized adaptive testing
CN: control normal
IRB: Institutional Review Board
LLM: large language model
MCI: mild cognitive impairment
MLP: multilayer perceptron
RoBERTa: Robustly Optimized BERT Pretraining Approach
WHO: World Health Organization
XAI: explainable artificial intelligence
