# Supplementary material for: Advancing Alzheimer Disease Prediction With Large Language Model–Based Linguistic Feature Analysis: Development and Validation Study
Source: JMIR Med Inform. 2026 May 28;14:e86965. doi: 10.2196/86965 (PMC13218719; doi:10.2196/86965)
Supplement: Multimedia Appendix 1 [file medinform-v14-e86965-s001.docx]

Appendix A. Prompt for Extracting Participant’s Description from Transcripts

The following is the prompt for speaker attribution, which aims to assigning parts of a transcript to the participant.

*As a speech and language therapist, your expertise is crucial for identifying language dysfunctions in individuals with mild cognitive impairment (MCI) and Alzheimer’s disease (AD). These dysfunctions often arise from compromised semantic and pragmatic processing abilities. One of the most used examination tools to elicit discourse is the Cookie Theft picture (CTP) from the Boston Diagnostic Aphasia Examination. The Cookie Theft experiment involves asking participants to describe a detailed scene of a cookie theft depicted in an image, aiming to assess their cognitive function and memory.*

*The given transcript records a dialogue, which is usually initiated by the examiner with questions like “What’s happening in that picture?” Then the participant will describe the picture of the cookie theft picture.*

*You are assigned to finish ONE task: remove the examiner’s part and leave the participant’s part in the transcript.*

*Format your final response with the original transcript and the modified transcript after removing the examiner’s part. You should answer with the following JSON format:*

{

“original transcript” : “I want you to take a look at this picture....”,

“Modified transcript”:“”

}

Appendix B. Prompt for AD Related Linguistic Feature Analysis by LLM and an Example Response

The following is the prompt for AD Related Linguistic Feature Analysis.

*As a speech and language therapist, your expertise is crucial for identifying language dysfunctions in individuals with mild cognitive impairment (MCI) and Alzheimer’s disease (AD). These dysfunctions often arise from compromised semantic and pragmatic processing abilities. One of the most used examination tools to elicit discourse is the Cookie Theft picture (CTP) from the Boston Diagnostic Aphasia Examination. The Cookie Theft experiment involves asking participants to describe a detailed scene of a cookie theft depicted in an image, aiming to assess their cognitive function and memory.*

*Provide scores across all features ranging from 1 (low level) to 7 (high level) and your comments on the transcript. Ensure that your comments include detailed explanations and evidence for each feature:*

1. *Readability: Readability refers to the ease with which a reader can understand a written text. Patients with Alzheimer’s disease often have difficulty producing sentences with high readability. A higher score of “Readability” refers to the text in the given transcript is easy to understand.*
2. *Fluency: The “fluency” of a written text refers to the smoothness, coherence, and ease with which a reader can process and understand the content. With Alzheimer’s disease, people might have trouble using verbs, auxiliary verbs, gerunds, and participles. They may also make more sentence fragments and use words that aren’t in the dictionary. A higher score of “Fluency” refers to the given transcript containing a consistent style and logical sequence.*
3. *Richness of Detail: Richness of detail refers to the amount of detail and information included in a description, allowing the reader to clearly visualize the scene or situation being described. Patients with Alzheimer’s disease often have difficulty providing descriptions with abundant detail. Their descriptions tend to be simpler and lack detailed observations and information. A higher score of “Richness of Detail” indicates that the description includes abundant detail and information.*
4. *Keyword Relevance: The participant’s description contains key terms that are relevant to the central elements of the scene in the Cookie Theft picture, such as “boy”, “cookie”, “cupboard”, “curtain”, “dishcloth”, “dish”, “exterior”, “girl”, “jar”, “kitchen”, “plate”, “sink”, “stool”, “water”, “window”, and “woman”. Patients with Alzheimer’s disease may have difficulty identifying or mentioning key elements of the scene. A lower frequency of relevant keywords in their descriptions may indicate cognitive decline. A higher score in “Keyword Relevance” suggests that the participant has identified and mentioned essential elements, which could correlate with better cognitive function.*

Here’s an example of a non-Alzheimer’s disease transcript:

“I see a mom doing the dishes and the water’s overflowing from the sink and she seems to be completely oblivious of it. The children in the picture who I presume are hers are climbing, one the boy is climbing on a or standing on a stool that’s about to fall over, putting his hand in the cookie jar on the top shelf and his sister or cousin or whoever the girl is is reaching up for a cookie. The window is open, there’s a pathway with garden on either side it looks like. There’s something else on the sink, I mean on the countertop dishes. And that’s the mother has an apron on, she is dressed in the Mrs. Beaver style with a very, with an apron and a nice dress and shoes and so forth and I don’t understand why she’s not turning the water off but that’s part of the deal and there’s curtains on the window and there’s panes in the window, small panes.”

Here’s a Alzheimer’s disease transcript:

“Well, the boy was taking some cookies from the cupboard up there, and he’s... the... Yeah, that there... stool, I call it. I don’t know what you call it. And what’s she doing? She’s asking for some cookies. Mother’s drying the dishes. And she spilled water all over the place, with the sink. She also has some cups and a dish sitting there. Her curtains, you can see. Your well. Ah, what else? That’s about it.”

Give your response with explanations and the corresponding scores. Here is the given transcript: {transcript}

Example Case Transcript: Adrsdt19, AD *“They’re making... Making a mess of the place. Oh. By not putting... by not... no needness. Yes, there’s a few accidents. The little boy is standing on a chair which is crooked. The lady has water running out of her sink. And... Towel in her hand which is dangerous. Cookies are coming down. The cookie jar is coming down. And the place is very pretty. Okay, good.”*

Response From LLM:

[Based on the provided transcript, I will evaluate the features mentioned above as follows:

1. Readability: The transcript has some pauses and hesitation, which may affect the overall readability score. The use of incomplete sentences and fragmented phrases also contributes to lower readability. Therefore, I would rate the readability score as 3.
2. Fluency: There are some breaks in fluency due to pauses and hesitations in the speech. The participant struggles to maintain a coherent and smooth flow of information. Therefore, the fluency score would be 2.
3. Richness of Detail: The description lacks detailed observations and sufficient information to create a vivid mental image of the scene. While some details are mentioned, overall, the description is relatively sparse. Therefore, the richness of detail score would be 3.
4. Keyword Relevance: The essential elements of the scene, such as the boy, chair, sink, cookies, and cookie jar, are mentioned in the description. However, some key terms are missing or not clearly identified. Therefore, I would rate the keyword relevance score as 4.

Overall, the given transcript demonstrates difficulties in readability, fluency, richness of detail, and keyword relevance, which are common features of language dysfunction in individuals with cognitive impairment like Alzheimer’s disease. This highlights the importance of further evaluation and intervention to address these language deficits and support communication abilities.]
